# Effectiveness of conservative interventions for sickness and pain behaviors induced by a high repetition high force upper extremity task

**DOI:** 10.1186/s12868-017-0354-3

**Published:** 2017-03-29

**Authors:** D. L. Xin, J. Hadrévi, M. E. Elliott, M. Amin, M. Y. Harris, A. E. Barr-Gillespie, M. F. Barbe

**Affiliations:** 1 Department of Surgery, University of Pennsylvania, 3450 Hamilton Walk, Philadelphia, PA 19104 USA; 2 Department of Public Health and Clinical Medicine, Occupational and Environmental Medicine, Umeå University, Umeå, Sweden; 3 Department of Neurosurgery, Thomas Jefferson University, 1020 Locust St., Philadelphia, PA 19107 USA; 4 Department of Anatomy and Cell Biology, Temple University School of Medicine, 3500 North Broad St., Philadelphia, PA 19140 USA; 5 College of Health Professions, Pacific University, 190 SE 8th Avenue, Hillsboro, OR 97123 USA

**Keywords:** Repetitive loading, Work-related musculoskeletal disorders, Upper extremity, Social interaction, Aggression, Mechanical hypersensitivity, Von Frey, Inflammation, Cytokines

## Abstract

**Background:**

Systemic inflammation is known to induce sickness behaviors, including decreased social interaction and pain. We have reported increased serum inflammatory cytokines in a rat model of repetitive strain injury (rats perform an upper extremity reaching task for prolonged periods). Here, we sought to determine if sickness behaviors are induced in this model and the effectiveness of conservative treatments.

**Methods:**

Experimental rats underwent initial training to learn a high force reaching task (10 min/day, 5 days/week for 6 weeks), with or without ibuprofen treatment (TRHF vs. TRHF + IBU rats). Subsets of trained animals went on to perform a high repetition high force (HRHF) task for 6 or 12 weeks (2 h/day, 3 days/week) without treatment, or received two secondary interventions: ibuprofen (HRHF + IBU) or a move to a lower demand low repetition low force task (HRHF-to-LRLF), beginning in task week 5. Mixed-effects models with repeated measures assays were used to assay duration of social interaction, aggression, forepaw withdrawal thresholds and reach performance abilities. One-way and two-way ANOVAs were used to assay tissue responses. Corrections for multiple comparisons were made.

**Results:**

TRHF + IBU rats did not develop behavioral declines or systemic increases in IL-1beta and IL-6, observed in untreated TRHF rats. Untreated HRHF rats showed social interaction declines, difficulties performing the operant task and forepaw mechanical allodynia. Untreated HRHF rats also had increased serum levels of several inflammatory cytokines and chemokines, neuroinflammatory responses (e.g., increased TNFalpha) in the brain, median nerve and spinal cord, and Substance P and neurokinin 1 immunoexpression in the spinal cord. HRHF + IBU and HRHF-to-LRLF rats showed improved social interaction and reduced inflammatory serum, nerve and brain changes. However, neither secondary treatment rescued HRHF-task induced forepaw allodynia, or completely attenuated task performance declines or spinal cord responses.

**Conclusions:**

These results suggest that inflammatory mechanisms induced by prolonged performance of high physical demand tasks mediate the development of social interaction declines and aggression. However, persistent spinal cord sensitization was associated with persistent behavioral indices of discomfort, despite use of conservative secondary interventions indicating the need for prevention or more effective interventions.

**Electronic supplementary material:**

The online version of this article (doi:10.1186/s12868-017-0354-3) contains supplementary material, which is available to authorized users.

## Background

Work-related repetitive strain injuries, also known as work-related musculoskeletal disorders (WMSDs), are a leading cause of chronic pain and physical disability worldwide [38], and are associated with a wide range of inflammatory and degenerative diagnoses [62]. Their etiology in the upper extremity is multifactorial, and includes physical, individual, job and social cultural factors [18, 33, 52]. Key physical risk factors include high repetition, high force, cumulative exposure and inadequate recovery times between exposures [33]. These disorders are associated with increased sickness absence, depression and chronic pain, and accounted for 32% of all lost work time in the US in 2015 [11, 23]. Biobehavioral mechanisms underlying work related depression and chronic pain changes are still under investigation [29], as are effective interventions [50, 58, 72].

Sickness behaviors, also known as Systemic Inflammatory Response Syndrome, are a constellation of physiological and behavioral responses that include depression, social withdrawal, allodynia and more [14, 73]. Sickness behaviors can be induced in animal models by administration of exogenous inflammatory cytokines either peripherally or centrally [48, 54]. Peripherally increased pro-inflammatory cytokines provoke central nervous system (CNS) neuroinflammation via several mechanisms: diffusion from the circulatory system into the brain through blood brain barrier deficient areas; active transport by endothelial cells; activation of blood brain barrier or endothelial cells that then activate neurons and glia; and retrograde transport of afferent nerve signals centrally [48, 54, 73]. This is relevant to human subjects with upper extremity WMSDs show increased serum levels of TNFalpha, IL-1beta and soluble receptors, IL-6 or macrophage inflammatory proteins [12, 36, 56, 63]. We postulate there is a link between elevated serum inflammatory cytokines and sickness behaviors occurring in subjects with WMSDs as prior studies have found associations between high job psychological strain and serum levels of C-reactive protein [28]. However, the role of inflammation and its negative effects on the CNS is an under-investigated topic in the clinical management of workers with increased sickness absences, depression or persistent pain.

We have developed a unique rat model of voluntary repetitive reaching and grasping lever pulling task in which prolonged performance of repetitive tasks induces exposure-dependent tissue injury, increased inflammation in involved musculoskeletal tissues that, when left untreated, drive degradative and fibrotic tissue changes and sensorimotor declines [1, 6, 31, 40]. Treatment of rats performing a high repetition high force (HRHF) task with ibuprofen or by moving the HRHF rats to an easier task attenuated local bone inflammatory and degradative responses [7, 24, 40], although we have yet to explore inflammatory cytokines in other tissues after these interventions. We hypothesize that these interventions should reduce task-induced serum inflammatory cytokine responses, for example. Surprisingly, ibuprofen treatment attenuated early but not long-term reflexive grip strength declines in our model [1, 44], leading us to hypothesize that CNS neuroinflammatory processes underlie the development, but not the maintenance of pain-related behaviors, as previously suggested from surgically-induced nerve injury studies [39, 57, 67]. This hypothesis has not been explored in reference to WMSDs; nor have we examined sickness behaviors or their treatment in our rat model.

Thus, we evaluated the effectiveness of conservative interventions (provision of ibuprofen or switching rats from the HRHF task to a reduced force and reach rate task for preventing: (1) declines in social interaction with a novel juvenile rat; (2) decreased forepaw withdrawal threshold; (3) decreased operant reaching and grasping abilities; (4) increased serum levels of inflammatory cytokines and chemokines; and (5) increased neuroinflammation in the median nerve, brain and spinal cord. Decreased social interaction and exploration are thought to be signs of depression in rats and was chosen as a measure of sickness behavior [71]. Ibuprofen was chosen as one intervention as it is a common nonsteroidal anti-inflammatory drug used world-wide by workers experiencing pain [2, 8, 15, 49]. Since long-term ibuprofen can have negative side effects [4], we also explored a nonpharmacological intervention of ergonomic task reduction.

## Methods

### Animals

Experiments were approved by the Institutional Animal Care and Use Committee and were in compliance with NIH guidelines for the humane care and use of laboratory animals. All rats were housed in an AAALAC-accredited animal facility in separate cages with a 12-h light:dark cycle and free access to water, and were provided with environmental enrichment in their home cages (chew toys and tunnels). All rats were handled at least 3 days per week by the same handlers to reduce investigator-induced stressors. Female Sprague–Dawley rats were used since human females have a higher incidence of WMSDs than males [16]. Rats were procured at 4–7 weeks of age and housed (while being handled until onset of experiments at 2.5 months of age (young adults). Rats were 3–7 months of age at completion. Behavioral procedures were conducted at the same times per day to minimize effects related to diurnal factors.

All but normal control rats were food-restricted to body weights of no less than 5% lower than age-matched normal controls to motivate interest in food reward pellets. Rats were weighed weekly and allowed to gain weight similarly across the course of the experiments, as they were young adults, as reported previously [7, 40]. In addition to food pellet rewards (a 1:1 mix of purified grain and banana 34 mg flavored pellets; Bioserve, NJ, USA), all rats received grain-based Purina rat chow daily (control rats also received the same number of food reward pellets as task rats). Rats were inspected weekly and again post-mortem for presence of illness or tumors to reduce confounders for serum cytokine increases (none were observed).

A total of 159 rats were used in this study. They included:

- Normal control rats (NC) euthanized at 1 week (n = 21) or at 19 weeks (n = 18) after onset of the experiment (the latter for euthanasia at matched time points as 12-week HRHF rats), with no training or task performance;
- Age-matched food-restricted control rats (FRC) euthanized at 1 week (n = 9) or at 19 weeks (n = 14) after onset of the experiment (the latter for euthanasia at matched time points as 12-week HRHF rats), with no training or task performance;
- Age- and weight-matched food-restricted rats that underwent training for 10 min/day for 6 weeks to pull to high force levels before euthanasia immediately post training (TRHF rats, n = 14);
- TRHF rats that received 40 mg/kg body weight doses of oral ibuprofen, daily, across the entire 6-week training period, and then euthanized post training (TRHF + IBU rats, n = 12);
- TRHF rats that rested after the training period until euthanasia at matched time points as 12-week HRHF rats (TRHF + Rest, n = 7).
- Untreated TRHF rats that then went on to perform a high repetition high force (HRHF) task for 6 or 12 weeks, untreated (6- and 12-week HRHF rats; n = 10 and 15, respectively);
- Untreated TRHF rats that then went on to perform a HRHF task for 4 weeks, before being administered liquid ibuprofen (Children’s Motrin Grape Flavored, Johnson & Johnson) in drinking water daily (40 mg/kg body weight) for 2 or 8 weeks, beginning in week 5, as described [24], while still continuing to perform the HRHF task (6- and 12-week HRHF + IBU rats; n = 12 and 11, respectively); and
- Untreated TRHF rats that then went on to perform a HRHF task for 4 weeks, that were moved to a low repetition low force (LRLF) task for 2 or 8 weeks, beginning in week 5, as previously described [7] (6- and 12-week HRHF-to-LRLF rats, n = 5 and 13, respectively).

### Behavioral apparatuses

Sixteen custom-designed behavioral apparatuses were used, as previously described [6, 13] and depicted [55]. Briefly, custom-designed force apparati were integrated into standard open field boxes placed into larger sound dampening boxes (Med Associates, St. Albans, VT). Each box had a portal located at the rats’ shoulder height, through which the rats reached through and pulled on a 1.5 mm metal bar, termed a force lever bar, positioned 2.5 cm outside of the chamber wall. This bar was attached to a miniature tension–compression load cell (Model LSB200, Futek Advanced Sensor Technology, Irvine, CA) interfaced with a strain-gauge amplifier (Model CSG110, Futek). The load cell signal was low pass filtered at 50 Hz before being sampled digitally at 100 Hz using Force Lever activity software (ENV-118 M, Product Number SOF-808, Med Associates) that allowed us to choose a set force level to which rats had to pull to receive a food reward. Auditory indicators (Med Associates) cued the animal to attempt a reach. The rats had to grasp the force lever and exert an isometric pull in the correct time frame and within target thresholds (defined further below). If these criteria were met within a 500 ms cueing period, a reward light came on signaling the arrival of a 45 mg purified formula food pellet (Bioserve, NJ) into a trough located at floor height for the animal to lick up.

### Training and task regimens

Training and task procedures were as previously described [6]. Briefly, all 159 rats were handled for 1 week prior to onset of experiments; this week was also the onset of food restriction in all but normal control rats. Subsets of rats were randomly chosen to serve as normal controls (n = 39) or food restricted only controls (n = 23) at this time point. The remaining rats (n = 97) were trained to pull a lever bar at high force levels during a 6-week initial training period of 10 min/day for 5 days/week. The target high force effort was 55% ± 5% of their mean maximum voluntary grasping force (110 ± 5.4 grams of force (gf); 1.078 Newton’s). Subsets of the trained rats were euthanized immediately post training, as either untreated trained only rats (TRHF rats, n = 14), trained only rats that rested for 12 weeks (TRHF + Rest, n = 7), or trained only rats that received 40 mg/kg body weight doses of oral ibuprofen, daily across the entire 6-week training period (TRHF + IBU rats, n = 12). Note, the post training time point is equal to 0 weeks of the HRHF task. The remaining 66 trained rats went on to perform a high repetition high force (HRHF) lever pulling task rats for 2 h/day and 3 days/week. This task was divided into 4, 0.5-h sessions separated by 1.5 h in order to avoid satiation. HRHF rats were cued to reach at a rate of 4 reaches/min and to grasp and hold a force lever bar for at least 15 ms, although a target of 300 ms was expected during the 500 ms cueing period, at a target force effort of 55 ± 5% of their mean maximum voluntary grasping force (1.078 Newton’s). Subsets of HRHF rats were randomly chosen to be untreated HRHF rats (n = 25), HRHF + IBU rats (n = 23), or HRHF-to-LRLF rats (n = 18), as described above. The ibuprofen treatment began in HRHF task week 5 [40]. HRHF-to-LRLF rats were moved to a low repetition low force task, beginning in week 5, that consisted of 2 reaches/min at 15% (0.29 Newton’s) of their maximum voluntary grasping force, for 2 h/day and 3 days/week [7]. Rats were not prevented from reaching at a higher or lower force than their target force; however, if they either undershot the minimum criterion, no food reward was delivered. Thus, the animals were allowed to self-regulate their participation in task performance, making these voluntary tasks.

### Determination of reach performance behaviors in task rats

Force lever data were recorded continuously during each task session for later calculation of the dependent variables (reach force and grasp time) via an automated script (MatLab; Mathworks, Natick, MA). Reach force was the mean recordable reach force (in Newton’s) applied to the force lever bar for all reaches across a given day. Grasp time was the mean average time (in seconds) the rats spent pulling on the lever bar for all recordable reaches. The mean grasp force and grasp time were calculated over the interval that ended when the force fell below 2.5% of baseline. From these data, the mean reach impulse (force × time) was calculated and reported for each individual rat by multiplying the mean reach force in Newton’s by the mean grasp time in seconds. Data for each variable was calculated on the last day of weeks 1, 3, 6 and 12. Week 1 was used as the baseline for reach performance variables since that was the first week rats actually performed the task regimens for 2 h/day, 3 days/week.

### Social interaction behaviors

Adult control, trained or task rats were placed into clean and clear plastic chambers with plastic bottoms and allowed to acclimate for 10 min. We then timed (in seconds) the duration of social exploration of the adult rat towards a novel juvenile rat (4–6 weeks of age) during the first 5 min (300 s total) following introduction of the juvenile rat. The following were considered as positive interactions with the juvenile rat: (1) sniffing of any kind (face-to-face or genitoanal), (2) crawling over or under, and (3) grooming of one another. Aggression by the adult rat towards the juvenile rat was also noted on incidence during this 5 min testing period, and included boxing, lateral displays, biting, wrestling and kicking. Such actions ended the social interaction session in accordance with our Animal Care and Use Committee guidelines, and the juvenile rat was removed from the cage immediately (thus, physical wounds were avoided). The person carrying out the testing was naive to group assignment. The mean incidence per testing session, normalized to number of rats tested, is reported. All animals were maintained in micro-filter topped cages in the housing room to preclude introduction of pheromone cues, and novel juvenile rats were brought into the behavioral testing room only prior to this testing for the same purpose. The number of novel juvenile rats used is not included in the final animal count as they were enrolled in other experiments post use.

### Forepaw withdrawal response threshold (mechanical allodynia)

Forepaw withdrawal behavioral responses to von Frey filaments (North Coast Medical, Morgan Hill, CA) were assayed as previously described [13]. The force of the smallest sized filaments (in grams) to which the rats withdrew their forepaw after stimulation was considered the response threshold. Forepaw von Frey withdrawal threshold data for the preferred reach limb only is reported. The person carrying out the testing was naive to group assignment.

### ELISA analysis of inflammatory cytokines and chemokines in serum and the median nerve

Following anesthesia using sodium pentobarbital (i.p., 120 mg/kg body weight), at 18 h after completion of the final task and behavioral testing sessions (to avoid exercise- or acute stress-induced serum cytokine/chemokine changes), blood was collected from all rats by cardiac puncture using a 23-gauge needle and centrifuged immediately at 1000×*g* for 20 min at 4 °C. Serum was collected and stored at −80 °C until analyzed for 15 cytokines and chemokines using a customized multiplex ELISA system (Aushon Biosystems Inc., Billerica, MA). Serum was assayed for 14 cytokines and chemokines: (a) five inflammatory cytokines (IL-1alpha and beta, IL-2, IL-4 and TNFalpha); (b) a proteic cytokine (IL-6); (c) an anti-inflammatory cytokine (IL-10); (d) a chemokine that activates/macrophages and T cells (interferon gamma, IFN-gamma); (e) a chemokine that stimulates differentiation and function of hematopoietic precursor cells from various lineages including macrophages (granulocyte macrophage–colony stimulating factor, GM–CSF); (f) two C–C motif inflammatory chemokines chemotactic for macrophages, neurotrophils and leukocytes [CCL3, also known as macrophage inflammatory protein 1 alpha (MIP1a); and CCL20, also known as MIP3a]; and (g) three C–X–C motif chemokines secreted by macrophages and mast cells [CXCL1, also known as Growth-Related Oncogene/Keratinocyte-derived Cytokine (Gro/KC); CXCL2, also known as MIP2; and CXCL3 (also known as Cytokine-Induced Neutrophil Chemoattractant-2 (CINC2)]. The array sensitivity of the serum analytes tested were (in pg/ml): CCL3 (0.8 pg/ml), CCL20 (1.6 pg/ml), CXCL1 (0.4 pg/ml), CXCL2 (0.2 pg/ml), CXCL3 (0.4 pg/ml), GM–CSF (3.1 pg/ml), IL-1alpha (1.5 pg/ml), IL-1beta (6.2 pg/ml), IL-2 (6.2 pg/ml), IL-4 (0.8 pg/ml), IL-6 (6 pg/ml), IL-10 (0.8 pg/ml), IFN-gamma (6.2 pg/ml) and TNFalpha (3.1 pg/ml). All samples were analyzed in duplicate in a blinded fashion, and batched as much as possible to reduce potential inter-assay variability. Data are presented as pg/ml serum.

The median nerve was collected from the forearm and palm, flash frozen, homogenized in previously described lysis buffer [5] using 0.2 ml microtissue grinders (Wheaton, Millville, NJ), and assayed similarly for IL-1beta, TNFalpha and IL-10. Data are presented as pg of cytokine protein to micrograms of total protein, determine using a bicinchoninic acid protein assay kit.

### Immunohistochemical analysis of cytokines in brains

Brains of rats were collected and examined immunohistochemically from six FRC, nine TRHF, ten 12-week HRHF, nine 12-week HRHF + IBU rats, and four HRHF-to-LRLF rats. For this purpose, rats were euthanized with an overdose of sodium pentobarbital (120 mg/kg body weight, serum collected for the analysis described above, followed by transcardial perfusion with 4% paraformaldehyde in 0.1 M phosphate buffer (pH 7.4). Brain tissues were collected, postfixed by immersion overnight, and stored in 30% sucrose for 3 days until cryosectioned into 20 µm coronal sections, and mounted on charged slides (Fisher Plus). Brain sections, on slides, were blocked with 4% goat serum in phosphate buffered saline (PBS) and incubated with the following primary antibodies: IL-1beta (Millipore/Chemicon, AB1832P; 1:250 dilution in PBS) and TNFalpha (Millipore/Chemicon, AB1837P; 1:250 dilution in PBS), for overnight at room temperature. After washing the sections with PBS, primary antibodies were visualized using appropriate anti-rabbit or anti-mouse secondary antibodies (from Jackson Immuno) conjugated to diaminobenzidene (DAB, brown for IL-1beta; combined with eosin counterstain) or Cy3 (red fluorescence for TNFalpha), diluted 1:250 in PBS, for 2 h at room temperature before washing. Slides were washed and then cover-slipped with 80% glycerol in phosphate buffer and viewed with Nikon microscopes (E800 and E1000) linked to a digital cameras (Q-Imaging Retiga camera) linked to a computer with image capturing software (Q-Capture) and image analysis software (Bioquant, Nashville, TN). Immunohistochemical variability was minimized by performing cytokine immunohistochemistry as a batched assay for each experiment.

Brain sections were examined for cytokine immunoreactivity in the following regions: ependymal, brain parenchyma surrounding ventricles, hypothalamus and anterior cingulate cortex. The percent area fraction of brain regions with cytokine immunoreactivity was quantified as previously described, in batched sets by the same individual who was blinded to rat treatment. Six measurements were made per section in 3 brain sections per rat, each section separated by 140 µm. Measurements were made using a set square area of 13.3 cubic microns available with the Bioquant image analysis system (Bioquant, Nashville, TN).

Preabsorption controls were also performed to demonstrate if the antibodies bound specifically to the antigen of interest. A two to ten fold excess of purified protein was pre-incubated with the matching antibody overnight at 4 °C (purified recombinant IL-1beta and TNFalpha proteins from Millipore were used), the mixture centrifuged, and then the pre-absorbed antibody supernatant was incubated with the brain sections (after pepsin and goat serum treatment) similarly to that described above, before washing and incubation with secondary antibodies. No labeling was observed in the tissues for any pre-absorbed antibody (data not shown). We also performed in which serum was substituted for the primary antibody, followed by secondary antibodies. No labeling was observed as a result of incubation of tissues with serum and then secondary antibodies alone (data not shown).

### Analysis of spinal cords for TNFalpha, GFAP, substance P and neurokinin 1 receptor

Spinal cords were examined immunohistochemically in 10 FRC, eight 12-week HRHF, six 12-week HRHF + IBU and six HRHF-to-LRLF rats. Cervical spinal cord segments were collected, processed and frozen sectioned, as described previously [26]. Spinal cord tissues were immunostained using the following primary antibodies: anti-TNFalpha (same antibody and dilutions as for brain), anti-glial fibrillary acidic protein (GFAP, a marker of activated astrocytes; Millipore, MAB360; 1:800 dilution in PBS), anti-substance P (Millipore/Chemicon AB1556, 1:250 dilution in PBS) and anti-neurokinin-1 receptor (NK-1R; Millipore/Chemicon AB5060, 1:1000 dilution in PBS), as previously described [26, 27]. Preabsorption controls or no primary antibody controls were performed as described above or as described previously [25, 31]. The percent area fraction of cervical spinal cord regions with immunoreactivity for each analyte was quantified, as previously described in detail [26, 27].

### Availability of data and materials

There is no code and no biological materials to share. All relevant raw data analyzed during the current study are available from the corresponding author on reasonable request.

### Statistical analyses

Results are expressed as mean and standard error of the mean (SEM). All statistical analyses and calculations are derived using the XLStat statistical software to assay for repeated measures behavioral data with unequal numbers per group, followed by the Tukey post hoc correction method for multiple comparisons. First, a mixed-effects model with repeated measures was used to determine effects of 5% food restriction on behaviors in FRC versus NC rats with free access to food across time, with the factors group and time in experiment (1 vs. 19 weeks). Mixed-effects models with repeated measures were then used for behavioral assays occurring across weeks with the factors group and week. Prism Graphpad statistical software was used to assay for serum and tissue data, followed by the Bonferroni or Tukey correction methods for multiple comparisons. Serum was first analyzed using the factors group and week to determine which had the highest induced levels, followed by analysis using the factors treatment and week to determine effects on selected individual serum cytokines. One-way ANOVAs were used to assess brain, spinal cord and median nerve cytokine and/or GFAP immunoexpression or levels between groups. Two-way ANOVAs were used to compare immunoexpression of substance P and neurokinin 1 receptor in the spinal cord using the factors treatment and week. For conciseness, details of significant ANOVA and posthoc results are presented in individual figure panels. Adjusted p values are reported, and an adjusted *p* value of <0.05 was considered significant for all analyses.

## Results

### Social interaction declines occurring in TRHF and HRHF rats are ameliorated with ibuprofen or move to a low demand task

**Fig. 1:**
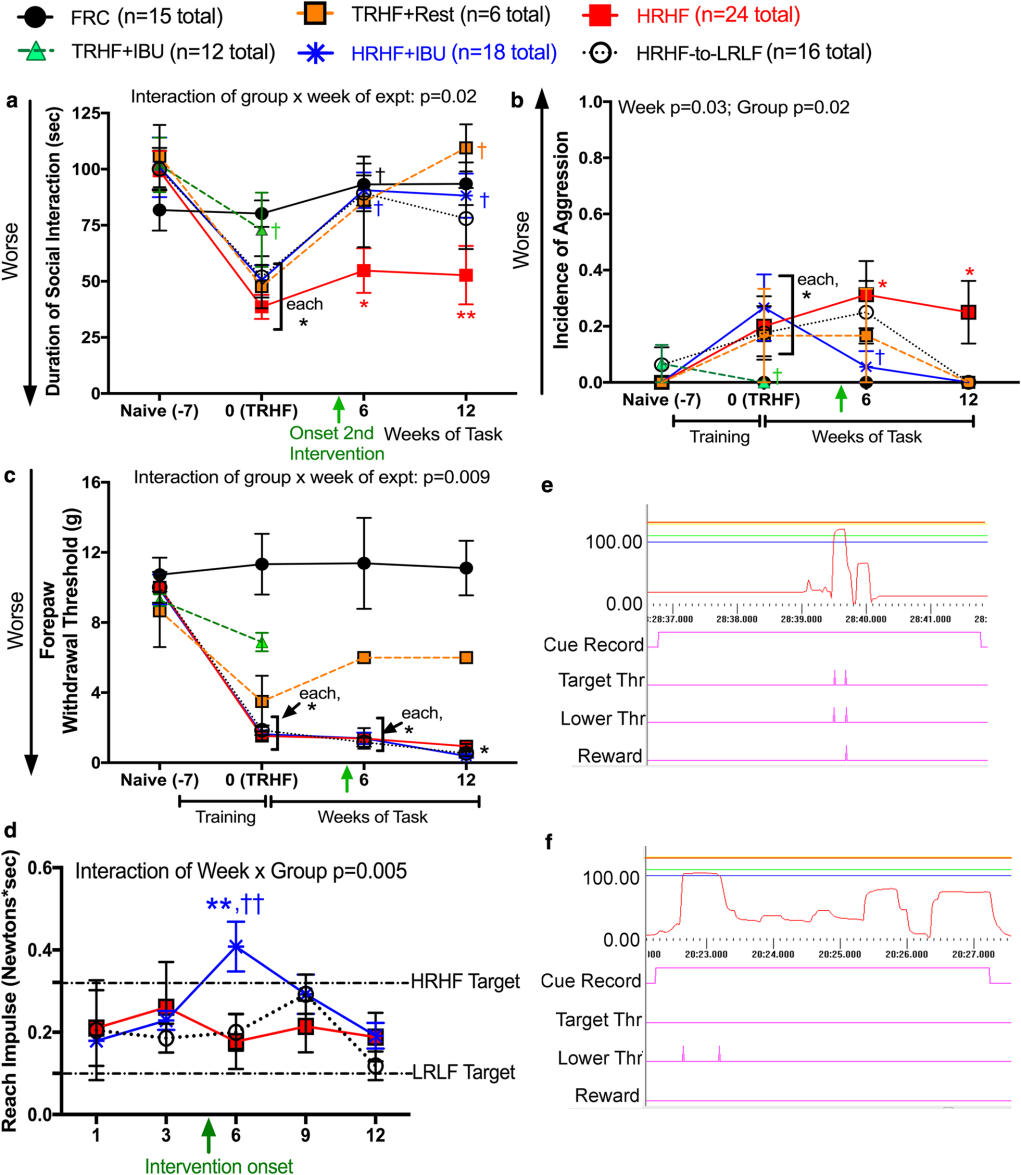
Duration of social interaction, aggression, forepaw withdrawal behavioral changes and reach performance abilities. **a** Duration of positive social interaction (in seconds) of adult rats with a novel juvenile rat during a 300 s observation period. **b** Incidence of aggression of the adult rat with a novel juvenile rat during social interaction period (which ended the test, thus lowering the duration of positive social interaction in **a**). **c** Forepaw withdrawal response to light probing of glabrous skin at midpoint of forepaw. Y-axis shows size of von Frey filament (in grams) required to elicit a withdrawal response (mean of 5 probings shown). **d** Reach impulse (Newton’s of reach force multiplied by grasp time in seconds). HRHF and LRLF targets are indicated by *dashed lines*. **e** Example of a successful reach. The *upper orange line* indicates the maximum force threshold, the *blue line* depicts the lower force threshold; the *green line* depicts the target force threshold; and the *pink line* above the *time marker* shows the force deflection. The *reward line* shows that a food reward was delivered to a trough for rats to lick up. **f** Example of three unsuccessful reaches in one 500 ms cueing cycle, in which all reaches were below the target threshold (*green line*); thus, no food reward was dispensed. Results of two-way ANOVAs for each behavioral parameter are shown. *p < 0.05 in **a**–**c**, compared to age-matched FRC rats; **p < 0.01 in **d**, compared to week 1 results of same group; ^†,††^p < 0.05 and p < 0.01, respectively, compared to age-matched untreated HRHF rats

The duration of social interaction with a novel juvenile rat declined precipitously with training to high force levels (TRHF rats and 0-week HRHF time point) and remained decreased in HRHF rats through week 12, compared to age-matched FRC rats (Fig. 1a). This response was ameliorated in TRHF + IBU rats, TRHF + Rest rats, 6-week HRHF + IBU and HRHF-to-LRLF rats, and 12-week HRHF + IBU rats, compared to age-matched untreated TRHF or HRHF rats (Fig. 1a). Duration of social interaction was near control levels in 12-week HRHF-to-LRLF rats, although not significantly improved from 12-week HRHF levels.

Aggression increased immediately post training, and in untreated 6- and 12-week HRHF rats, compared to FRC rats (Fig. 1b). Ibuprofen attenuated this increase in TRHF + IBU rats and 6-week HRHF + IBU rats, compared to untreated TRHF and HRHF rats, respectively (Fig. 1b). The 12-week TRHF + Rest, 12-week HRHF + IBU and HRHF-to-LRLF rats showed similar incidence of aggression as FRC rats (Fig. 1b).

### Mechanical allodynia present in TRHF and HRHF rats is attenuated in TRHF + IBU rats, but not by secondary intervention task rats

The size of von frey filament needed to elicit a forepaw withdrawal response declined precipitously after training in TRHF and 0-week HRHF rats, and remained decreased in HRHF rats through week 12 (indicative of mechanical allodynia), compared to age-matched FRC rats (Fig. 1c). This response recovered towards control levels in TRHF + IBU rats and in TRHF + Rest rats at 6 and 12 weeks of rest, although neither were significantly different from untreated trained or task rats. In contrast, HRHF + IBU and HRHF-to-LRLF rats showed similar low threshold levels as untreated HRHF rats at both time points assayed (Fig. 1c).

### Reduced HRHF task performance abilities were only partially rescued by ibuprofen

The mean reach impulse of untreated HRHF rats was lower than target levels throughout all weeks of task performance (Fig. 1d). Figure 1e depicts a successful reach occurring within the correct time frame and to the target force levels, and dispensal of a food reward. Figure 1f depicts a representative and often occurring unsuccessful reach by a 6-week HRHF rat. The rat pulled several times within one cueing period of 500 ms, yet each reach failed to meet the target force threshold, suggestive of discomfort. As a consequence, no food reward was dispensed during this cueing cycle. Ibuprofen treatment improved the mean reach impulse in HRHF + IBU rats in week 6, compared to their week 1 levels and untreated HRHF rats (Fig. 1d). However, this improvement was transient and their mean reach impulse declined to untreated levels by week 12, suggestive of discomfort. HRHF-to-LRLF rats showed an appropriate decrease in reach impulse by week 12 to low force reach impulse levels (Fig. 1d).

### Training to high force (TRHF) and extended HRHF task performance increased inflammatory cytokines in serum and brain; changes attenuated by the interventions

No differences in serum cytokine/chemokine levels were observed in the NC versus FRC rats at 1 week after the onset of experiments, or at matched time points as 12-week HRHF rats (Additional file 1: Figure S1A and B). In contrast, two way ANOVAs using the factors cytokine and group showed that IL-1beta, IL-6 and TNFalpha increased the most with training or task performance, compared to the other cytokines and chemokines assayed (Additional file 1: Figure S1).

**Fig. 2:**
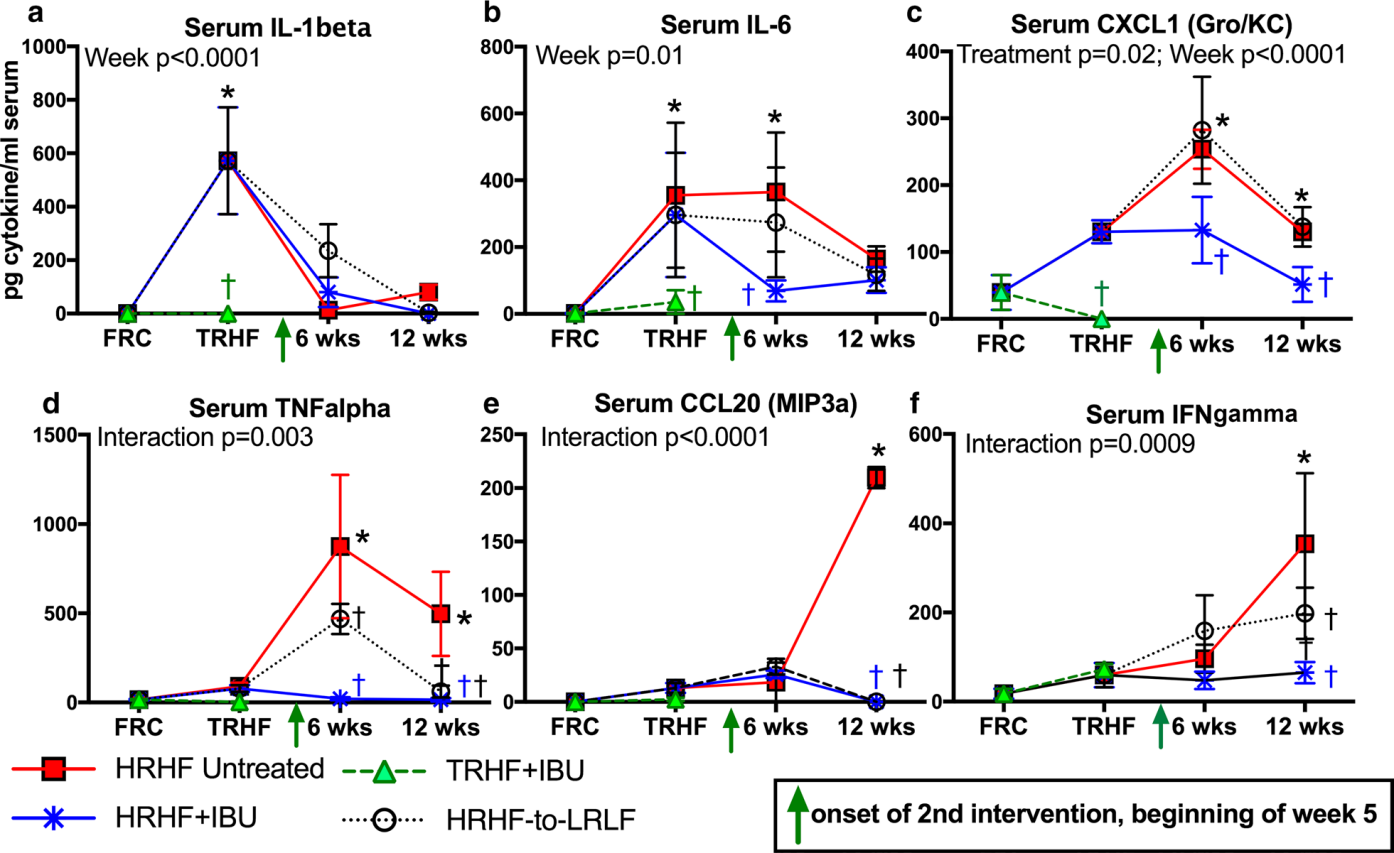
Serum levels of several cytokines and chemokines, assayed using multiplex ELISA. Results of two-way ANOVAs for each cytokine are shown in* each panel*, using the factors treatment group and week of experiment. Serum levels of **a** IL-1beta, **b** IL-6, **c** CSCL1 (Gro/KC), **d** TNFalpha, **e** CCL20 (MIP3a), and **f** IFNgamma are shown for each group, across weeks of training and task performance, as indicated. *p < 0.05, compared to food restricted control (FRC) rats euthanized after 1 week of food restriction; ^†^p < 0.05, compared to age-matched untreated TRHF or untreated HRHF rats

**Fig. 3:**
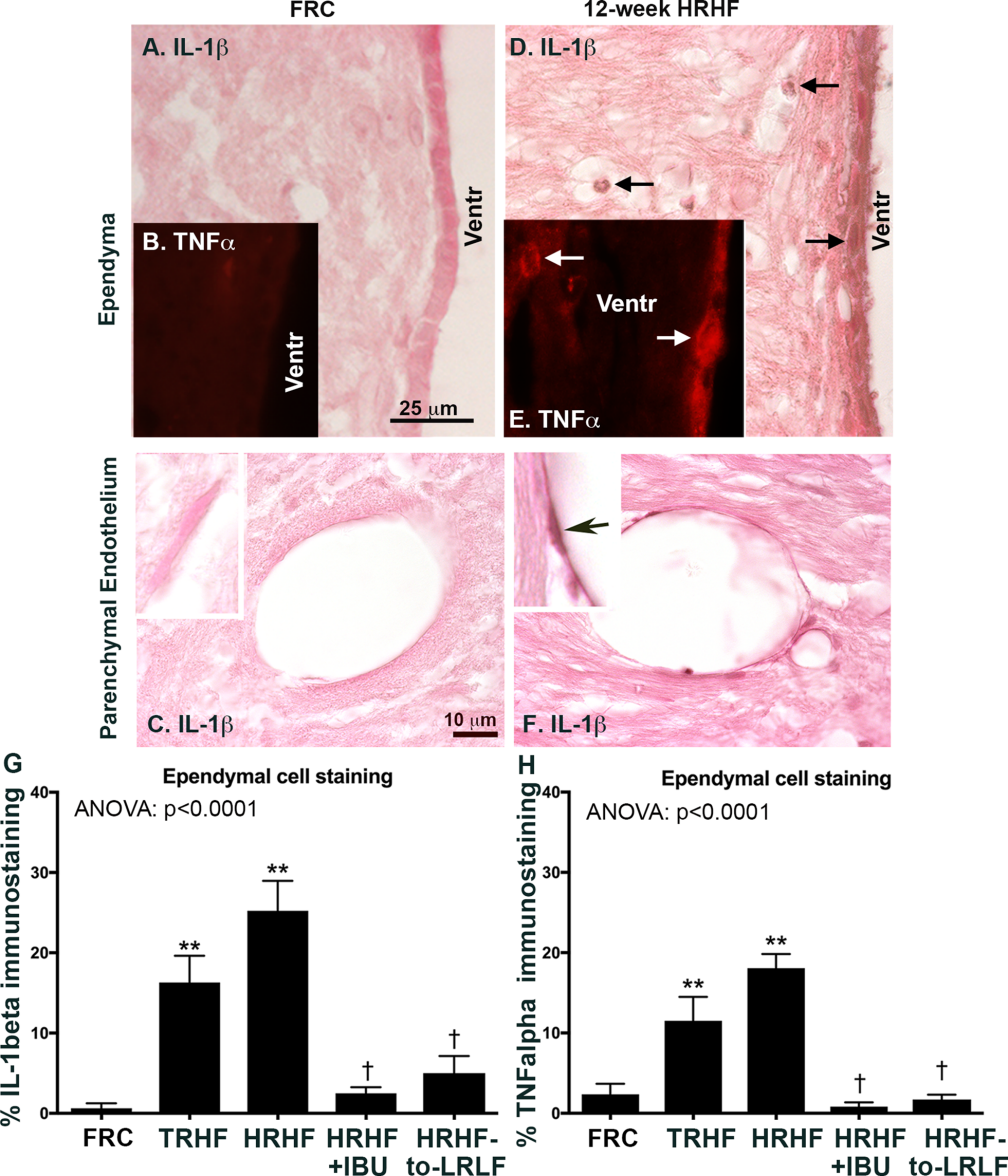
Inflammatory cytokine immunoreactivity in brain ependymal and endothelial cells. **A**–**C** There was an absence of IL-1beta and TNFalpha in these cell types in FRC rat brains. **D**–**F** IL-1beta and TNFalpha immunoreactivity increased in these cell types in 12-week HRHF rats. In **D** and **E**, *arrows* indicate positively stained ependymal cells surrounding the ventricle as well as small stained glial-like cells within the brain parenchyma. In **F**, *inset* shows IL-1beta stained endothelial cell. **G**, **H** Percent area with IL-1beta and TNFalpha immunoreactivity, respectively, in ependymal cells. Results for one-way ANOVAs are shown. **p < 0.01, compared to FRC rats; ^†^p < 0.05, compared to untreated HRHF rats

Follow-up two-way ANOVAS on individual cytokines revealed that the initial training increased serum levels of IL-1beta and IL-6 in TRHF rats, compared to FRC rats (Fig. 2a, b). CXCL1 (Gro/KC) also increased with training, although not significantly (Fig. 2c). These training-induced increases were attenuated in TRHF + IBU rats, compared to untreated TRHF rats (Fig. 2a–c). Serum IL-1beta levels resolved over time in HRHF task rats to FRC levels. IL-6 remained increased in 6-week HRHF rats, compared to FRC rats, although it resolved towards control levels by week 12 (Fig. 2b). Serum CXCL1 (Gro/KC) and TNFalpha levels were increased in 6- and 12-week HRHF rats above FRC levels (Fig. 3C, D); both were significantly attenuated in HRHF + IBU rats (Fig. 2c, d). 12-week HRHF-to-LRLF rats showed reduced levels of TNFalpha, compared to untreated HRHF rats, although CXCL1 remained increased increased in this group, compared to FRC rats (Fig. 2c, d). By week 12 of HRHF task performance, serum levels of CCL20 (MIP3a) and IFNgamma were also increased above control levels (Fig. 2e, f). Both secondary interventions successfully attenuated these increases by week 12 to control levels (Fig. 2e, f). The 12-week TRHF + Rest rats also showed control levels of inflammatory cytokines and chemokines (data not shown).

Brains were collected and examined for IL-1beta and TNFalpha immunoreactivity. In FRC rats, an absence or only low levels of immunoreactivity were observed (Fig. 3A–C, G, H). In contrast, 12-week HRHF rat brains showed increased immunoreactivity for both cytokines in ependymal cells surrounding all ventricles, small glial-like cells adjacent to the ventricles, endothelial cells (Fig. 3D–F), and choroid plexus (not shown). The endothelial cells were scattered throughout the brain. No increased immunoreactivity for IL-1beta or TNFalpha were observed in the hypothalamus of any group, although low levels of IL-1beta staining was observed in the CA3 region of the hippocampus in 3 of 8 of the 12-week HRHF rats (data not shown). Quantification of IL-1beta and TNFalpha immunoreactivity in ependymal cells showed increases in TRHF and 12-week HRHF rats, compared to FRC rats, and significant attenuation of each in 12-week HRHF + IBU and HRHF-to-LRLF rats, compared to untreated HRHF rats (Fig. 3G, H). Cytokine immunostaining was also decreased in TRHF + IBU rat brains, compared to untreated TRHF rats (data not shown).

### HRHF task performance increased inflammatory cytokines in the median nerve, increases attenuated by the secondary interventions

**Fig. 4:**
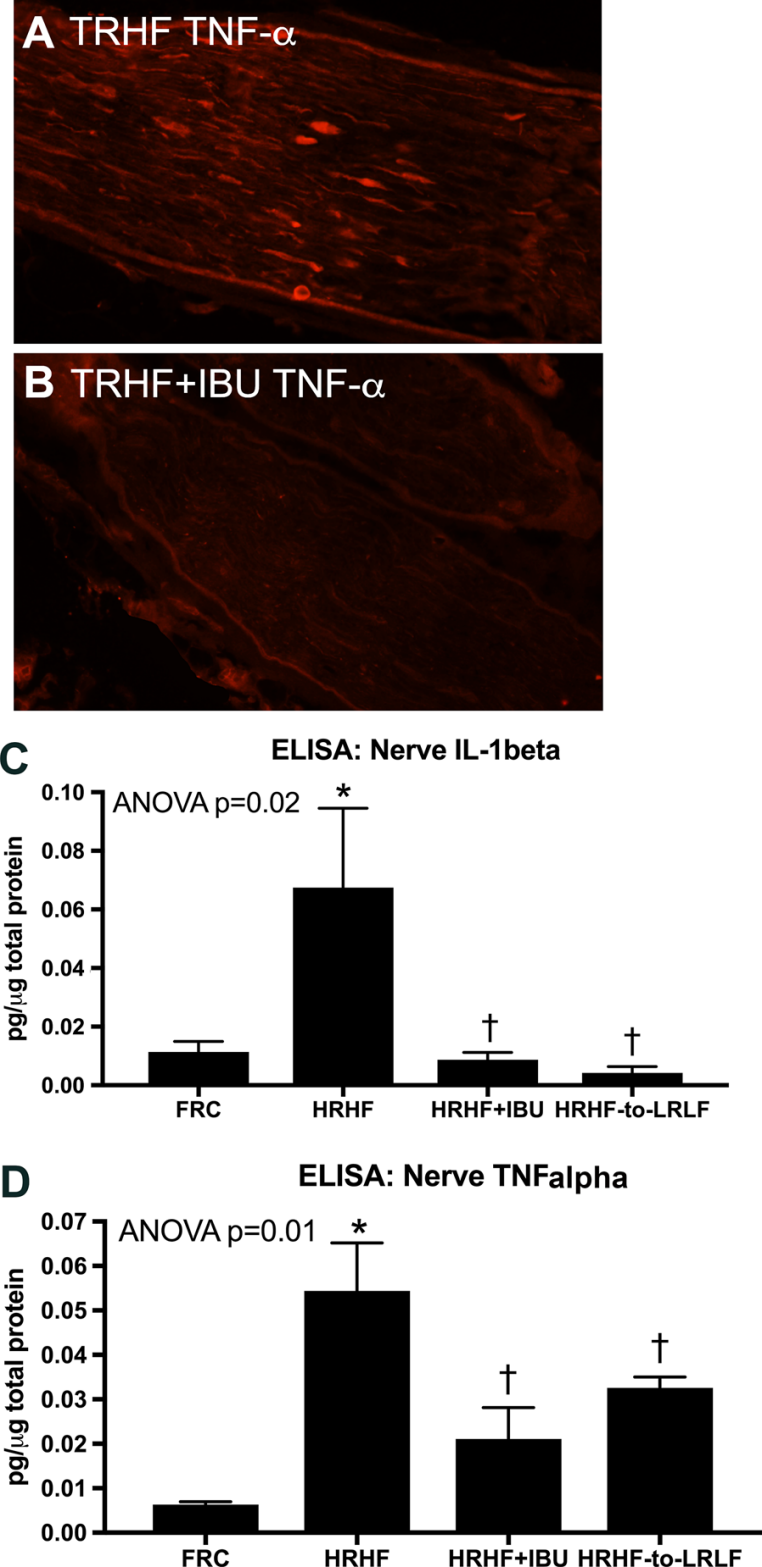
Inflammatory cytokines in the median nerve. **A**, **B** TNFalpha immunoreactivity in the median nerve at the level of the wrist of TRHF and TRHF + IBU rats, respectively. Images were taken with a 40× objective. **C**, **D** IL-1alpha and TNFalpha levels in the median nerve, respectively, assayed using ELISA. Results for one-way ANOVAs are shown. **p < 0.01, compared to age-matched untreated FRC rats; ^†^p < 0.05, compared to age-matched untreated HRHF rats

In the median nerve at the level of the wrist, increased immunoexpression of TNFalpha was observed in TRHF rats, but not in TRHF + IBU rats (Fig. 4A vs. B). ELISA analysis of the median nerve revealed increased IL-1beta and TNFalpha in HRHF rats, compared to FRC rats, and decreased levels in 12-week HRHF + IBU and HRHF-to-LRLF rats (Fig. 4C, D). There were no significant differences in IL-10 across the groups (data not shown).

### HRHF task performance increased inflammatory cytokines, GFAP, substance P and NK-1 in spinal cord dorsal horns, increases that the secondary interventions failed to fully attenuate

**Fig. 5:**
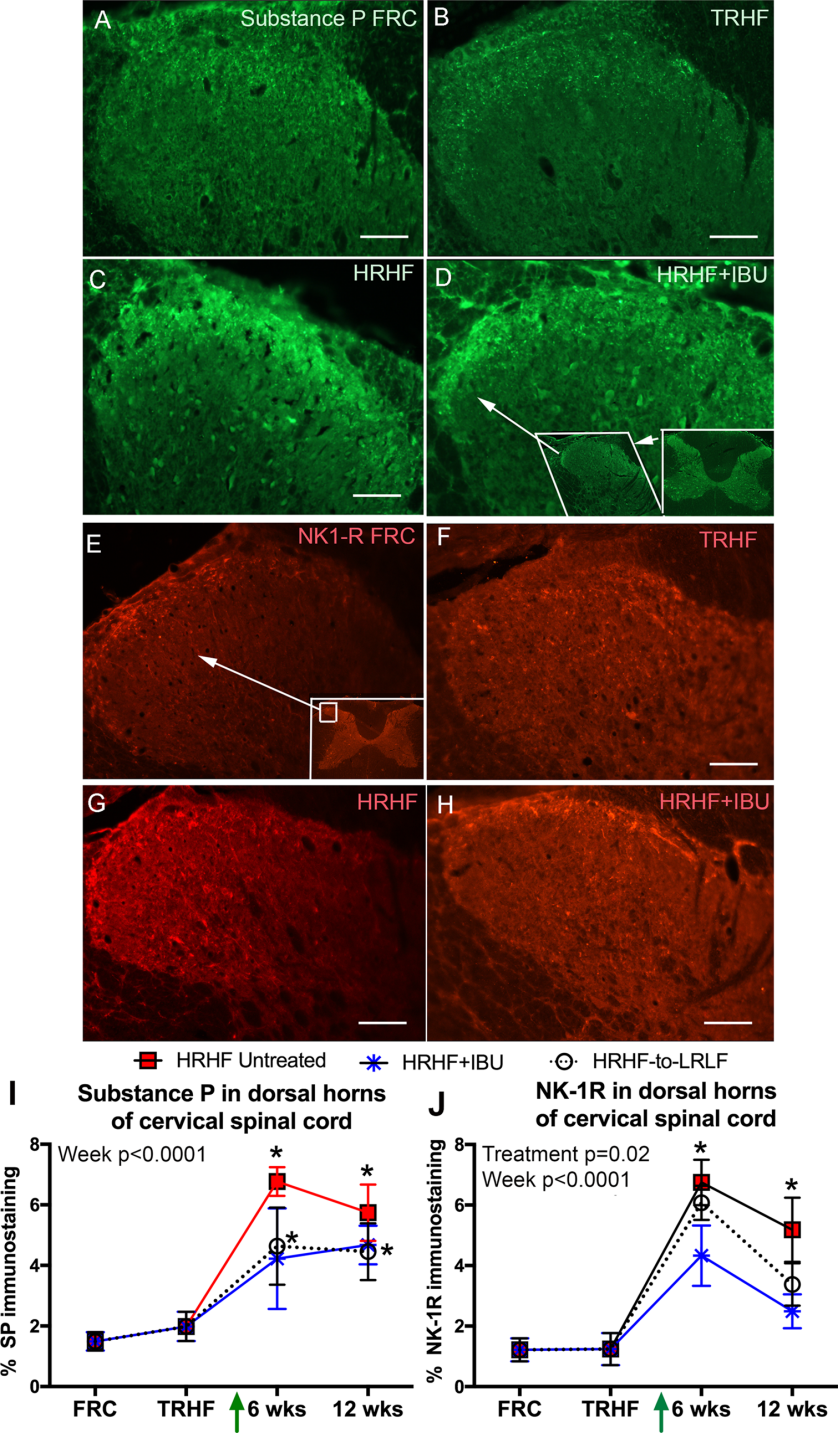
Substance P and NK-1R immunostaining in superficial lamina of dorsal horns of spinal cords at cervical 7 segmental level. **A**–**D** Representative images of dorsal horns showing punctuate substance P immunofluorescence (*green*) staining distributed across entire zone, medial to lateral (*right* to *left side*, respectively, in *each panel*). 12-week HRHF and HRHF + IBU rats show increased immunoexpression laterally. *Insets* in **D** shows location of substance P immunostaining at low power. *Scale bars* 50 μm, and applies to all panels except* insets*. **E**–**H** Dorsal horns showing neurokinin-1 receptor (NK-1R) immunostaining (*red*).* Inset* in **E** shows localization of NK-1R immunostaining at low power. **I**, **J** Quantification of percent area with immunofluorescence staining for substance P and NK-1R, respectively, in C7 dorsal horn superficial lamina. Results for two-way ANOVAs are shown. *p < 0.05, compared to age-matched untreated FRC rats

In dorsal horns of cervical spinal cord segments, increased immunoexpression of substance P and its main receptor, neurokinin-1 receptor (NK-1R), was observed in 6- and 12-week HRHF rats, compared to FRC rats (Fig. 5). Such spinal cord changes were not observed in TRHF rats (Fig. 5B, F, I, J). Substance P immunostaining remained increased in 6- and 12-week HRHF + IBU and HRHF-to-LRLF rats, compared to FRC rats (Fig. 5I). NK-1R immunostaining was partially attenuated (although not to control levels) in 12-week HRHF + IBU and HRHF-to-LRLF rats (Fig. 5J).

**Fig. 6:**
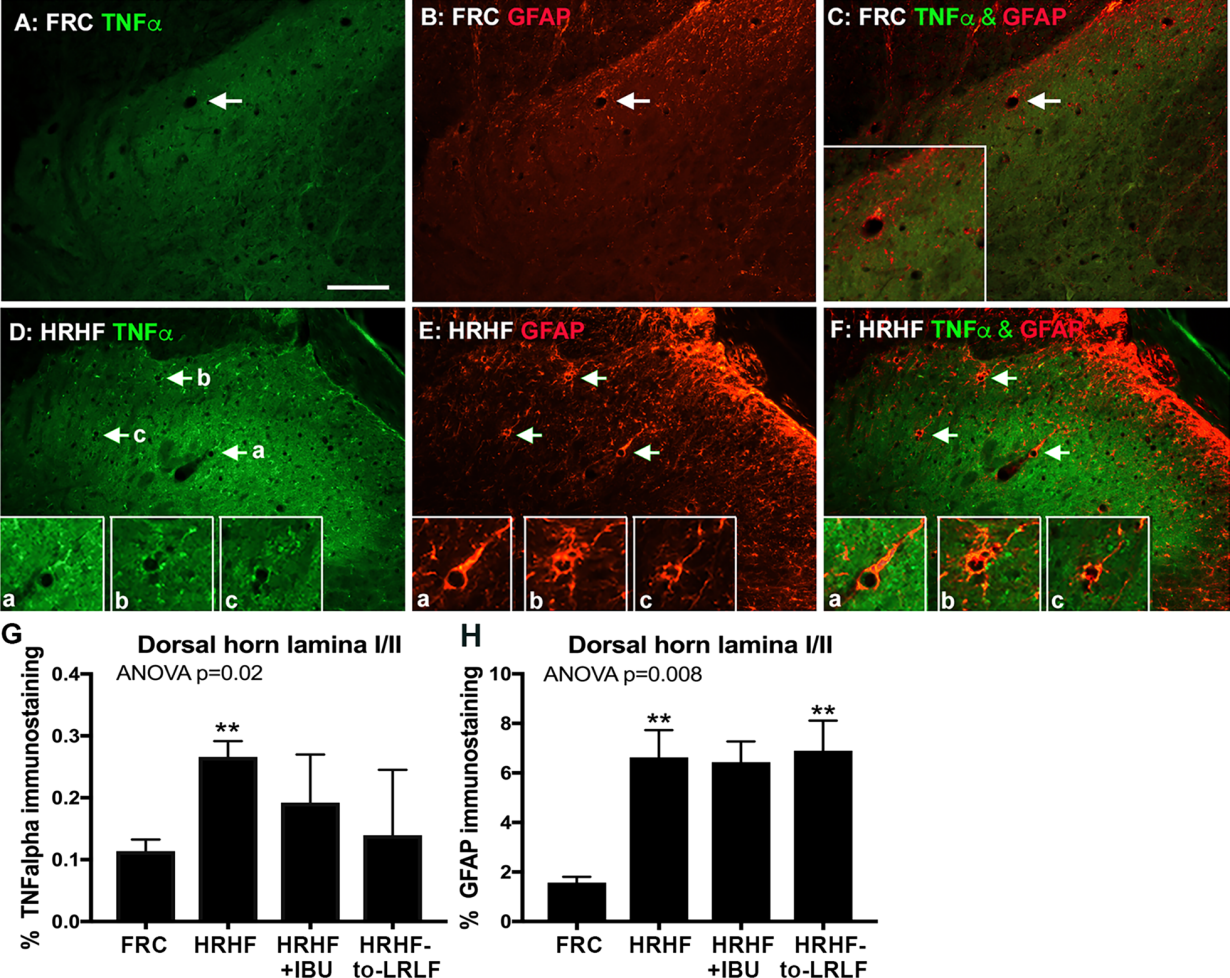
TNFalpha and GFAP immunostaining in the superficial lamina (lamina I and II) of dorsal horns of the spinal cord at cervical 7 segmental level. **A**–**C** Representative immunoreactivity in FRC rat for TNFalpha, GFAP, or both together. *Arrow* indicates same cells in *each panel*, showing a lack of overlap between TNFalpha and GFAP. *Scale bars* 50 μm, and applies to all other panels except* insets*. **D**–**F** Representative immunoreactivity in 12-week HRHF rat for TNFalpha, GFAP, or both together. *Arrows* and lettering *a*–*c* indicate cells shown enlarged in* insets* of *each panel* and depicts cells with TNFalpha and GFAP co-localization. **G**, **H** Quantification of percent area with TNFalpha and GFAP immunostaining. **p < 0.01, compared to food restricted control (FRC) rats

Based on these results, we explored possible spinal cord sensitization changes further by examining cervical spinal cord segments of control and task rats for TNFalpha increases and astrocyte activation. In superficial lamina of dorsal horns of cervical spinal cord segments, increased immunoexpression of TNFalpha was observed as increased punctate staining in 12-week HRHF rats, compared to FRC rats (Fig. 6D vs. A). Increased immunoexpression of GFAP was observed in this same region in 12-week HRHF rats, compared to FRC rats (Fig. 6E, H vs. B, H). Some TNFalpha-positive cell profiles co-localized with the GFAP immunoexpression (Fig. 6C, F and insets). These increases were not significantly attenuated by either secondary intervention (Fig. 6G, H).

**Fig. 7:**
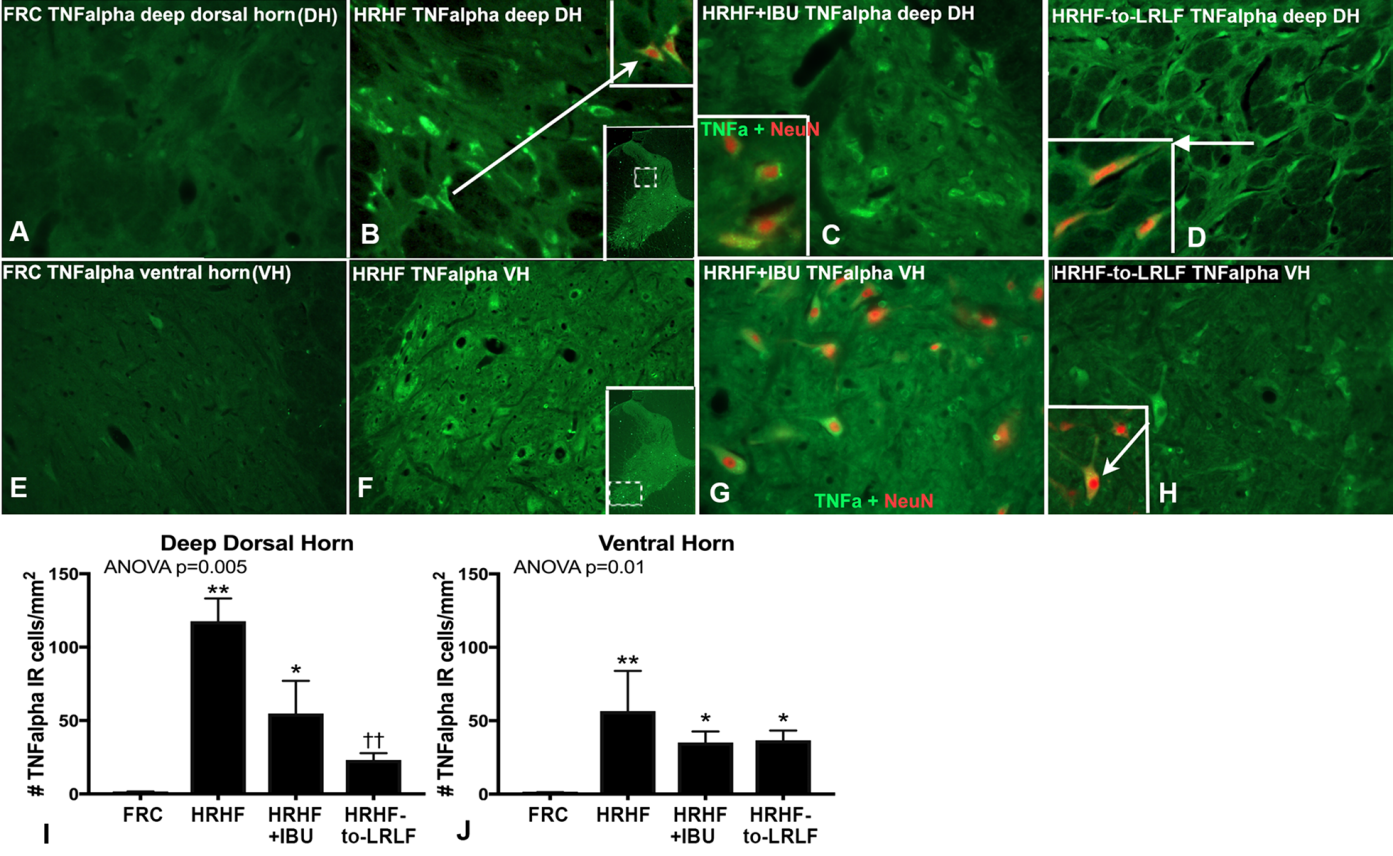
TNFalpha immunostaining (*green*) in dorsal horn deep lamina (DH) and ventral horns (VH) of the spinal cord at cervical 7 segmental level. NeuN, a nuclear stain, shown in *red*. **A**, **E** Representative immunoreactivity in FRC rat. **B**, **F** Representative immunoreactivity in untreated 12-week HRHF rats. *Arrow* in **B** indicates same cells as in *upper inset*, showing TNFalpha in NeuN stained neurons. *Lower insets* shows areas of assay for dorsal horn deep lamina and ventral horns, respectively. **C**, **G** Representative immunoreactivity in 12-week HRHF + IBU rat. *Inset* in **C** shows TNFalpha in NeuN stained neurons. **D**, **H** Representative immunoreactivity in 12-week HRHF-to-LRLF rat.* Insets* show TNFalpha staining in NeuN stained neurons. **I**, **J** Quantification of number of TNFalpha immunostained cells per mm^2^. **,*p < 0.05 and p < 0.01, compared to food restricted control (FRC) rats; ^††^p < 0.01, compared to age-matched untreated HRHF rats

Increased TNFalpha immunoexpression was also observed in deeper dorsal horn lamina and in the ventral horns of cervical spinal cord segments of 12-week HRHF and HRHF + IBU rats, compared to FRC rats (Fig. 7). The TNFalpha immunostaining in these regions was within NeuN immunopositive neurons (Fig. 7C, D, H insets, G). The TNFalpha immunoexpression in deeper dorsal horn lamina was significantly reduced in 12-week HRHF-to-LRLF rats, compared to untreated HRHF rats (Fig. 7I). TNFalpha immunostaining in ventral horn neurons remained increased in both secondary intervention groups, compared to FRC rats, and was not significantly reduced from untreated HRHF rat levels (Fig. 7D, H, J).

## Discussion

We show here for the first time in an operant model of work-related musculoskeletal disorders that extended performance of a high demand reaching and grasping task induced aggression and social interaction declines, concomitant with chronic brain neuroinflammatory changes. We also observed persistent increases in serum levels of several inflammatory cytokines and chemokines, and increased TNFalpha in the median nerve. The responses were attenuated by ibuprofen treatment (at prophylactic and secondary administration time points) and an ergonomic task reduction intervention. These findings novelly suggest that chronic inflammatory responses induced by prolonged performance of high physical demand tasks mediate the development and maintenance of subsets of sickness behaviors. However, HRHF task-induced pain related behaviors persisted, even after the treatments, perhaps because spinal cord neuroinflammation and neurotransmitter changes were not effectively reduced by these secondary interventions.

Human workers exposed to repeated workplace exposures to hand intensive tasks at high force and activity levels, have increased risk of new-onset pain [34, 37]. Human workers with WMSDs are also at risk for developing increased depression, aggression, chronic pain and reduced work performance abilities [23, 29, 58, 72]. Thus, behavioral findings (Fig. 1) from our rat model match the human condition. Epidemiological studies show that high psychological job demands and low job control are risk factors for developing upper limb WMSDs [9, 47, 51]. Our findings here also indicate that training to high physical force levels induces social interaction declines, aggression and pain-related behaviors. The improvement in TRHF + Rest rats, compared to untreated HRHF rats, further supports continued high force work as an inducing physical risk factor. Prophylactic ibuprofen treatment provided during training was also effective, indicating that inflammatory mechanisms are involved in the development of these behavioral declines. Both secondary interventions ameliorated incidence of aggression and improved duration of social interaction. However, neither attenuated the forepaw mechanical allodynia, and secondary ibuprofen treatment only transiently improved reach impulse abilities. Surgically induced nerve injury studies have shown that inflammatory cytokine increases within injured nerves are responsible for the development of pain-related behaviors, but not their maintenance [39, 57, 67]. From this, we suggest that provision of secondary interventions beginning in task week 5 was too late to rescue pain-related behaviors as nerve TNFalpha increases had already occurred (Fig. 4).

Human subjects with upper limb WMSDs of duration similar to this current study (≤12 weeks) have increased pain symptoms that correlate with increased serum TNFalpha, MIP1alpha and MIP1beta [12, 56, 64]. These were cross-sectional studies, and their findings differed from others examining serum cytokines in human subjects with MSDs of shorter or longer duration, in which either only soluble receptors of the IL-1 family or IL-6 were detected (≤1 month) or no cytokines were detected in serum at the time of median nerve decompression surgery [32, 46, 63]. A recent systematic review suggests the need for longitudinal and broader biomarker studies [35]. Therefore, we expanded beyond our past study of HRHF rats in which we examined only 3 cytokines in serum (IL-1alpha, IL-1beta and TNFalpha) at two time points [7], to examine 14 cytokines/chemokines across the 4.75 month span of this experiment. We observed considerable differences in the systemic inflammatory response over time. The initial training induced increased levels of IL-1beta and IL-6 (Fig. 2). Then, serum levels of IL-1beta resolved immediately post training, IL-6 levels resolved by week 12, CXCL1 and TNFalpha increases began in week 6, and by HRHF task week 12 there were increases in CXCL1, TNFalpha, CCL20 and IFNgamma. Thus, the time of serum collection affects the outcome in chronic studies. Each treatment (prophylactic ibuprofen, rest, and secondary interventions) successfully attenuated the increases in serum inflammatory cytokines (with the exception of CXCL1 levels which remained elevated in HRHF-to-LRLF rats), in parallel with decreases in aggression and improvement in social interaction time, the latter suggestive of an association between these findings.

Presence of tissue damage activates the immune system to induce local release of inflammatory cytokines and other mediators which can overspill into the circulatory system to drive widespread activation of defense pathways, such as the sickness behavior pathways [10]. The median nerve may be one source of the circulating TNFalpha (Fig. 4). We have previously reported median nerve injury in HRHF task rats in the form of myelin degradation and reduced nerve conduction velocity [13]. We have also shown increased muscle and bone levels of inflammatory cytokines in 12-week HRHF rats [6], and that their levels in bone are reduced by the secondary ibuprofen and ergonomic task reduction treatments [7, 24, 40]. Central brain production of inflammatory cytokines, such as the increased immunoexpression of IL-1beta and TNFalpha in ependymal and endothelial cells (Fig. 3) may also be maintaining their increase in serum in a bi-directional manner [70].

Known mechanisms by which peripheral pro-inflammatory cytokines provoke CNS neuroinflammation include diffusion from the circulatory system into the brain through blood–brain-barrier-deficient brain areas, active transport into the CNS by endothelial cells, and/or activation of blood brain barrier cells that then activate neurons, astrocytes or microglia [48, 54, 73]. The increased inflammatory cytokine immunoexpression in ependymal cells supports a diffusion mechanism in our model, while the increased immunoexpression of cytokines in endothelial cells suggests activated endothelial cell-to-astrocyte cell signaling. Our behavior and brain immunoexpression findings are in line with others showing reduced social interaction and increased aggression after exogenous administration of TNFalpha or IL-1beta [69, 75], and involvement of ependymal, endothelia and astrocytic cells in sickness behaviors [19, 66], respectively. Both secondary interventions successfully attenuated serum and brain inflammatory cytokine increases, social behavior declines and aggression, supporting an involvement of inflammatory cytokines in their development and maintenance as shown previously in models of other disorders [20, 42, 60]. Other labs have also shown that the development of such behaviors can be prevented or reversed by treatments with NSAIDS or more specific COX 1/2 inhibitors [21, 53, 68]. We add here that removal of the inducing factor (the high force task) using rest or an ergonomic task reduction, also successfully reduced serum and brain inflammatory responses and ameliorated the social interaction declines.

Increased inflammatory cytokines in injured peripheral nerves are known to induce spinal cord central sensitization, including increased inflammatory cytokines, substance P and NK-1R immunoexpression in spinal cord astrocytes, microglia and neurons [26, 43, 65]. The systemic inflammatory responses may also be contributing to spinal cord sensitization [30]. Central sensitization responses in the spinal cord are known to contribute heavily to persistent and chronic pain behaviors [41, 74]. Our findings of persistent reach performance declines in HRHF rats shown here and previously [44], as well as forepaw mechanical allodynia in treated task animals, concomitant with persistent spinal cord sensitization, may explain epidemiological reports of failure of many common secondary interventions (including ibuprofen) in attenuating pain in subjects with chronic MSDs [17, 58, 61]. The effectiveness of multifaceted interventions or other anti-inflammatory drugs should be explored, as discussed further below.

While the strengths of this study include a longitudinal study in age-matched animals, the use of an operant model, and the ability to examine both peripheral and central neural tissues, it is still nevertheless a rat model. Translation of some aspects of the study needs to be performed in humans with upper extremity work-related injuries. Also, multiple molecules or pathways may be activated and thereby serve as contributors to the observed sensorimotor declines. Ibuprofen reduces COX and prostaglandin E_2_ production and pain effectively, but not leukotriene B4 production [59]. Leukotrienes can contribute to nociceptive-related responses and spinal cord sensitization changes following peripheral nerve injury [3, 22, 45]. Broader anti-inflammatory drugs may be needed for more effective treatments of repetitive injury induced pain related behaviors.

## Conclusions

Performance of a high demand work task at high reach rate and grasping force levels induced increased inflammatory cytokines in serum as well as several peripheral and central neuroinflammatory responses. The peripheral inflammatory responses appeared to alter social interaction behaviors via humoral-to-brain neuroinflammation mechanisms, and to increase pain-related behaviors via peripheral nerve-to-spinal cord sensitization mechanisms. The social interaction declines and increased aggression were effectively reduced by common interventions (rest, ibuprofen and ergonomic task reduction). However, persistent behavioral indices of discomfort were associated with persistent spinal cord sensitization, indicating the continued need for prevention or more effective treatments.

## Additional file

**Additional file 1: Fig. S1** **.** Serum cytokines in TRHF and HRHF rats, with 3-month old controls (A), or 7-month old controls that are age-matched to 12-week HRHF rats (B). Two-way ANOVA results (with the factors group and cytokine) are shown. Symbol < indicates p < 0.05, compared to groups as indicated in the key.

## Abbreviations

ANOVA: analysis of variance
CCL20: C–C motif L20 inflammatory chemokine, also known as macrophage inflammatory protein 3 alpha (MIP3a)
CCL3: C–C motif L3 inflammatory chemokine, also known as macrophage inflammatory protein 1 alpha (MIP1a)
CINC2: cytokine-induced neutrophil chemoattractant-2
CNS: central nervous system
CXCL1: C–X–C motif L1 chemokine, also known as growth-related oncogene/keratinocyte-derived cytokine (Gro/KC)
CXCL2: C–X–C motif L2 chemokine, also known as macrophage inflammatory protein 2 (MIP2)
CXCL3: C–X–C motif L3 chemokine, also known as cytokine-induced neutrophil chemoattractant-2 (CINC2)
DAB: diaminobenzidene
FRC: food-restricted control rats
GFAP: glial fibrillary acidic protein
GM–CSF: granulocyte macrophage–colony stimulating factor
gf: grams force
Gro/KC: growth-related oncogene/keratinocyte-derived cytokine
HRHF: high repetition high force
HRHF rats: untreated HRHF rats
HRHF + IBU: HRHF rats treated with ibuprofen
HRHF-to-LRLF: HRHF rats that were moved to a low repetition low force (LRLF) task after week 4
IL-1beta: interleukin-1beta
IL-6: interleukin 6
LRLF: low repetition low force task
MIP1a: macrophage inflammatory protein 1 alpha
MIP2: macrophage inflammatory protein 2
ms: milliseconds
NC rats: untreated normal control rats
NK-1R: neurokinin-1 receptor
PBS: phosphate buffered saline
s: seconds
SEM: standard error of the mean
TNFalpha: tumor necrosis factor alpha
TRHF: rats trained to high force task levels
TRHF + IBU: TRHF rats treated with ibuprofen
WMSDs: work-related musculoskeletal disorders
