# Supplementary figures and images for: Effectiveness of conservative interventions for sickness and pain behaviors induced by a high repetition high force upper extremity task
Source: BMC Neurosci. 2017 Mar 29;18:36. doi: 10.1186/s12868-017-0354-3 (PMC5371184; doi:10.1186/s12868-017-0354-3)

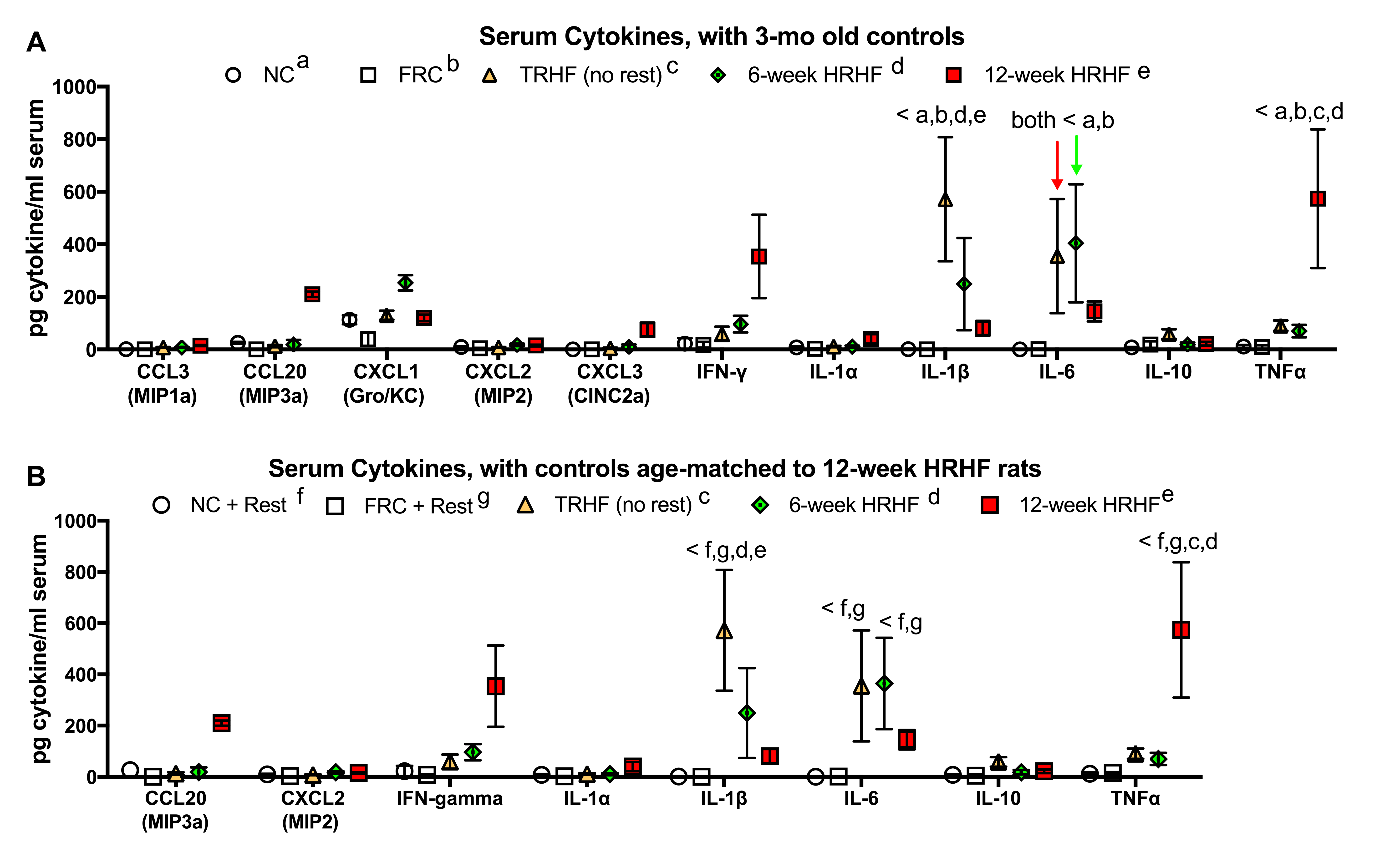

Supplement: Supplementary file 1 — Additional file 1: Fig. S1 . Serum cytokines in TRHF and HRHF rats, with 3-month old controls (A), or 7-month old controls that are age-matched to 12-week HRHF rats (B). Two-way ANOVA results (with the factors group and cytokine) are shown. Symbol < indicates p < 0.05, compared to groups as indicated in the key. [file 12868_2017_354_MOESM1_ESM.tif]
